# Expression and functional analysis of the nobiletin biosynthesis-related gene *CitOMT* in citrus fruit

**DOI:** 10.1038/s41598-020-72277-z

**Published:** 2020-09-17

**Authors:** Mao Seoka, Gang Ma, Lancui Zhang, Masaki Yahata, Kazuki Yamawaki, Toshiyuki Kan, Masaya Kato

**Affiliations:** 1 Graduate School of Integrated Science and Technology, Shizuoka University, 836 Ohya, Suruga, Shizuoka, 422-8529 Japan; 2 Department of Bioresource Sciences, Faculty of Agriculture, Shizuoka University, 836 Ohya, Suruga, Shizuoka, 422-8529 Japan; 3 School of Pharmaceutical Sciences, University of Shizuoka, 52-1 Yada, Suruga-ku, Shizuoka, 422-8526 Japan

**Keywords:** Plant sciences, Secondary metabolism

## Abstract

Nobiletin, a polymethoxy flavone (PMF), is specific to citrus and has been reported to exhibit important health-supporting properties. Nobiletin has six methoxy groups at the 3′,4′,5,6,7,8-positions, which are catalyzed by *O*-methyltransferases (OMTs). To date, researches on OMTs in citrus fruit are still limited. In the present study, a novel OMT gene (*CitOMT*) was isolated from two citrus varieties Satsuma mandarin (*Citrus unshiu* Marc.) and Ponkan mandarin (*Citrus reticulata* Blanco), and its function was characterized in vitro. The results showed that the expression of *CitOMT* in the flavedo of Ponkan mandarin was much higher than that of Satsuma mandarin during maturation, which was consistent with the higher accumulation of nobiletin in Ponkan mandarin. In addition, functional analysis showed that the recombinant protein of CitOMT had methylation activity to transfer a methyl group to 3′-hydroxy group of flavones in vitro. Because methylation at the 3′-position of flavones is vital for the nobiletin biosynthesis, CitOMT may be a key gene responsible for nobiletin biosynthesis in citrus fruit. The results presented in this study will provide new strategies to enhance nobiletin accumulation and improve the nutritional qualities of citrus fruit.

## Introduction

Flavonoids are a group of secondary metabolites that include more than 10,000 kinds of derivatives^1^. According to their structure, flavonoids are divided into several subgroups, including flavanones, flavones, flavonols, flavanols, isoflavones, and anthocyanidins^2^. Flavonoids are biosynthesized from *p*-coumaroyl-CoA and three molecules of malonyl-CoA in the phenylpropanoid synthetic pathway^3^. In flavonoid biosynthesis, several modification reactions, such as hydroxylation, methylation, glycosylation, and malonylation, occur to produce individual flavonoids^4^. The position and the number of hydroxyl groups, which are prone to undergo methylation or glycosylation, vary greatly among different flavonoids^5^.

Methylation of oxygen (O-methylation), nitrogen (N-methylation) and carbon (C-methylation) is a universal process critical to all organisms^6^. The biosynthesis of *O*-methylated flavonoids is catalyzed by *O*-methyltransferase (OMT) using S-adenosylmethionine (SAM) as a methyl group donor to methylate the flavonoid hydroxy groups^7^. Plant OMT genes are categorized into two types, Types I and II, based on their molecular weight, amino acid sequence and bivalent ion dependency. Type I OMTs (caffeic acid OMT: COMT) are ion-independent enzymes with a molecular weight of 38–43 kDa. COMTs act on a wide range of substrates, such as myoinositol, chalcones, scoulerine, caffeic acid, 5-hydroxyferulic acid, caffeoyl CoA ester and 5-hydroxyferuloylester^8–10^. Type II OMTs (caffeoyl-CoA OMT: CCoAOMT) are ion-dependent enzymes involved in the monolignol biosynthesis with the molecular weight of 23–27 kDa. CCoAOMTs act on a narrow range of substrates, which act only on 5-hydroxyferuloyl CoA and caffeoyl CoA^11,12^.

It is well known that flavonoids play important roles in plants, such as inflorescence pigments^13^, signaling factors^14^, antioxidant^13,15^, antifungal^12^ and anti-insect^16^. In addition, flavonoids are important for human health due to their various bioactivities. Recently, it was reported that flavonoids possess anti-cancer^17,18^ and anti-allergy^19^ activities in in vitro experiments. Moreover, flavonoids have been found to exhibit anti-inflammatory^20,21^, anti-obesity^22^, and neuroprotective properties^23^ in animal models.

Nobiletin (3′,4′,5,6,7,8-hexamethoxyflavone), a polymethoxy flavone (PMF), is abundant in citrus flavedo (Supplementary Fig. S1)^24^. In recent years, the roles of nobiletin in human health have been investigated extensively, and it was suggested that nobiletin was beneficial to human health through its various activities, such as anti-depressant^25^, anti-allergy^26^, anti-pigmentation of skin^27^, and inhibition of heart failure progression^28^. Currently, the neuroprotective effect of nobiletin has attracted increasing attentions, and nobiletin has been expected to be a potential neuroprotectant for the treatment of cerebral ischemia–reperfusion injury^29^, Alzheimer’s disease, and Parkinson’s disease^30^. Therefore, the development of functional foods or supplements, which are rich in nobiletin, may be of great importance for human health.

In nature, nobiletin specifically accumulates in citrus fruits, and its content varies greatly among different citrus species. Nobiletin was markedly accumulates in species of the *Acrumen* and *Aurantium* sections, but not in the *Fortunella* and *Poncirus* species^24^. In particular, nobiletin is abundant in the flavedos of *Acrumen* species, such as Dancy tangerine (*C. tangerina*) and Ponkan mandarin (*C. reticulata*). Recently, the identification and functional characterization of OMTs have been reported in several plants species^4,31,32^. However, studies on OMTs in citrus fruits are still limited, and the nobiletin biosynthetic pathway in citrus remains to be elucidated. In this study, we investigated flavonoid accumulation in two citrus varieties, Satsuma mandarin (*Citrus unshiu* Marc.), which accumulates a low level of nobiletin, and Ponkan mandarin (*Citrus reticulata* Blanco), which accumulates a high level of nobiletin. Moreover, a novel *O*-methyltransferase gene *CitOMT* was isolated and its functions were investigated in vitro. The results showed that recombinant protein of CitOMT, which was found to perform methylation the 3′-position hydroxyl groups of flavones, was a key gene for the biosynthesis of nobiletin in citrus fruit. This study will contribute to the cultivation of high-quality citrus fruits that are rich in nobiletin and the production of supplements and nutrient-rich health foods.

## Results

### Flavonoids accumulation in citrus flavedo

**Figure 1:**
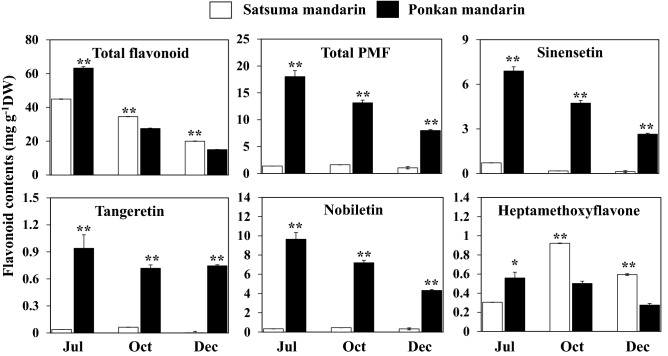
Flavonoid accumulation in the flavedos of Satsuma mandarin and Ponkan mandarin during fruit maturation. The total flavonoid content is the sum of identified flavonoids (narirutin, naringin, hesperidin, poncirin, isorhoifolin, rhoifolin, sinensetin, nobiletin, tangeretin and heptamethoxyflavone). Total PMF content is the sum of sinensetin, nobiletin, tangeretin and heptamethoxyflavone. Columns and bars represent the means ± SE (n = 3), respectively. The significant difference between Satsuma mandarin and Ponkan mandarin is indicated by asterisks (*P < 0.05, **P < 0.01).

In this study, the accumulation of flavonoids was investigated in the flavedos of Satsuma mandarin ‘Miyagawa-wase’ and Ponkan mandarin ‘Ohta Ponkan’ at three development stages: immature stage (July), transition stage (October), and mature stage (December). In the flavedos, four flavanones (narirutin, naringin, hesperidin, and poncirin), two flavones (isorhoifolin and rhoifolin), and four PMFs (sinensetin, nobiletin, tangeretin, and heptamethoxyflavone) were detected in Satsuma mandarin and Ponkan mandarin. As shown in Fig. 1, total flavonoid content decreased in Satsuma mandarin and Ponkan mandarin during fruit maturation. The total flavonoid content in Ponkan mandarin at the mature stage decreased to approximately one quarter of that at the immature stage. In Satsuma mandarin, the total flavonoid content at the mature stage decreased to approximately half of that at the immature stage. In addition, the flavonoid composition in the flavedos was different between Satsuma mandarin and Ponkan mandarin. During fruit maturation, Satsuma mandarin accumulated high levels of flavanones, which accounted for more than 85% of the total flavonoids, whereas low PMFs, which accounted for less than 6% of the total flavonoid content at the mature stage. In contrast, Ponkan mandarin accumulated higher levels of PMFs than Satsuma mandarin during the maturation. At the mature stage, PMFs accounted for more than 53% of the total flavonoid content in flavedo of Ponkan mandarin. In Ponkan mandarin, the major PMF is nobiletin, followed by sinensetin, tangeretin, and heptamethoxyflavone. During the fruit maturation, the contents of nobiletin, tangeretin, and sinensetin in Ponkan mandarin were much higher than those in Satsuma mandarin, whereas the content of heptamethoxyflavone in Ponkan mandarin was lower than that in Satsuma mandarin (Fig. 1).

### The isolation of *CitOMT*

In this study, we performed a Blast search in the Citrus clementina v.10 genome databases (https://www.phytozome.net/) using the sequence of ROMT-9 as a query, which has been reported to have strict specificity for the 3′-hydroxy group of flavonoids^33^. One OMT gene (Ciclev10020814m.g) was identified in the citrus genome database. We isolated the full-length CDS of *OMT* (*CitOMT*) from Satsuma mandarin (LC516612) and Ponkan mandarin (LC616611) using the primers designed according to the sequences obtained from the citrus genome database (Supplementary Table S1). The full-length nucleotide sequence of *CitOMT* in the two varieties contained 1,101 bp, encoding a putative protein of 366 amino acids with a predicted molecular weight of 40.0 kDa. The similarity of the deduced amino acid sequences between Satsuma mandarin and Ponkan mandarin was 99.5%. The deduced amino acid sequence of CitOMT shared more than 99% similarity with other citrus species, such as *Citrus aurantium* (putative caffeic acid *O*-methyltransferase: ADK97702.1) and *Citrus sinensis* (caffeic acid 3-*O*-methyltransferase: XP_006478090.1). The amino acid sequence of CitOMT also shared more than 82% similarities with non-citrus species, such as *Ricinus communis* (caffeic acid 3-*O*-methyltransferase: XP_002525818.1) and *Ziziphus jujuba* (caffeic acid 3-*O*-methyltransferase: XP_015878697.1.).

**Figure 2:**
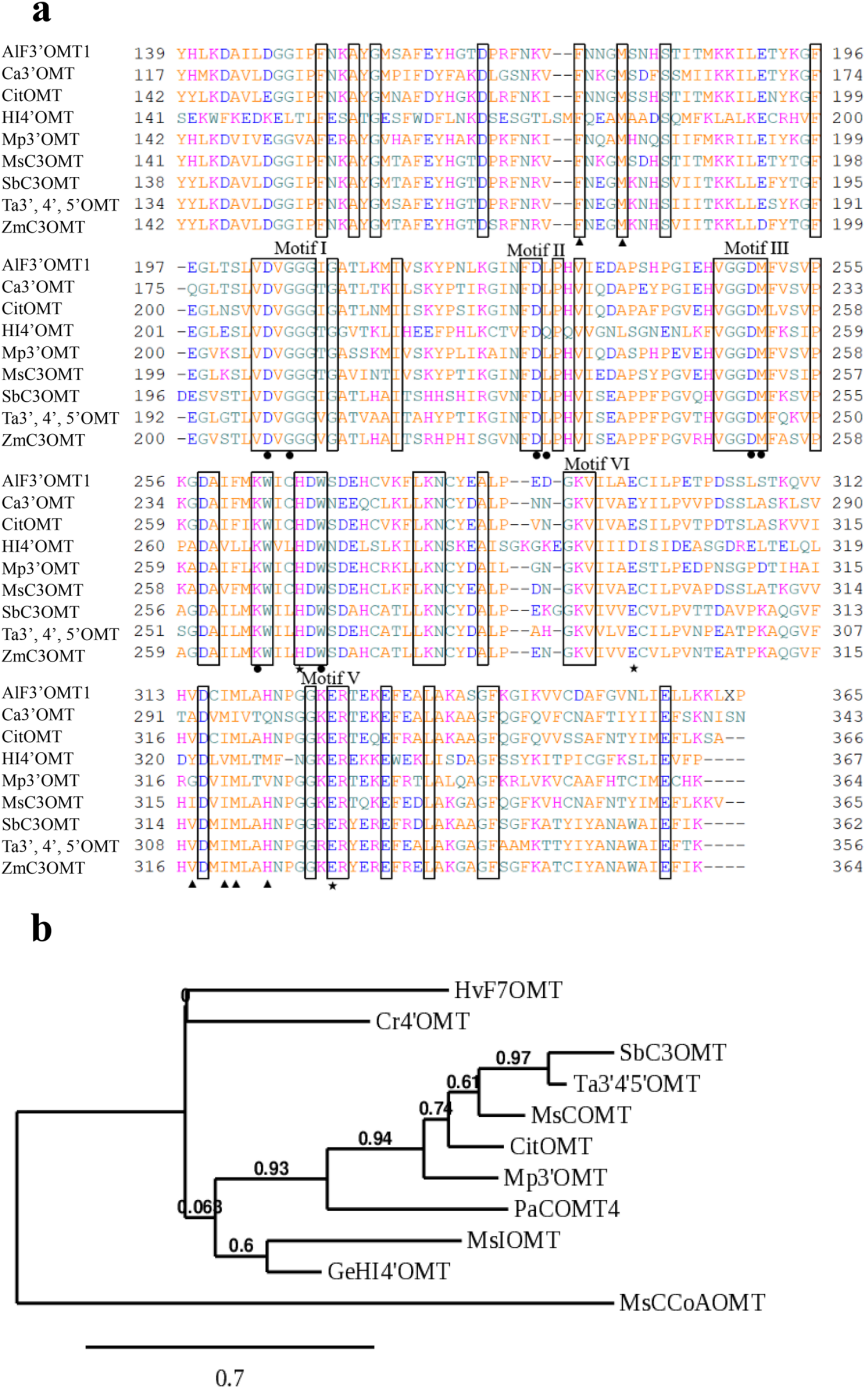
Multiple sequence alignment (**a**) and phylogenetic analysis (**b**) of CitOMT with other plant OMTs. The amino acid sequences of *C. reticulata* (CitOMT, LC516611), *Arabidopsis lyrata* subsp. lyrate (AlF3′OMT1, XP_020871511.1) *Chrysosplenium americanum* (Ca3′OMT, AAA80579), *Glycyrrhiz aechinata* (HI4′OMT, AB091684), *Mentha* × *piperita* (Mp3′OMT, AAR09601), *Medicago sativa* (MsC3OMT, ACY06328), *Sorghum bicolor* (SbC3OMT, AAL57301), *Triticum aestivum* (Ta3′,4′,5′OMT, ABB03907), and *Zea mays* (ZmC3OMT, ACG37598.1) are used in multiple sequence alignment. The same amino acids are shown in the rectangle. Residues involved in SAM binding (filled circles), substrate binding (filled triangles), and catalysis (★) were shown. These markers are shown at the bottom of the sequence. The five motifs are the relative positions of the OMT consensus regions (I–V)^5^. The amino acid sequences of *Catharanthus roseus* (Ca4′OMT, AAR02419.1), *Glycyrrhiz aechinata* (GeHI4′OMT, AB091684), *Hordeum vulgare* (HvF7OMT, CAA54616), *Medicago sativa* (MsCCoAOMT; AAC28973.1, MsCOMT; AAB46623, MsIOMT; MSU97125), *Mentha* × *piperita* (Mp3′OMT, AAR09601), *Sorghum bicolor* (SbC3OMT, AAL57301), *Triticum aestivum* (Ta3′,4′,5′OMT, ABB03907), *Plagiochasma appendiculatum* (PaCOMT4, KY977690) are used for phylogenetic tree analysis.

Multiple amino acid sequence alignment of CitOMT with other plant OMTs was conducted using GENETYX (Fig. 2a). CitOMT showed conserved motifs (Motif I-V), which may be involved in interactions with the cofactor SAM^5,34^. The amino acid residues (His-270, Glu-298 and Glu-330 in CitOMT) are known as the catalytic residues in MsIOMT^6^. A phylogenetic tree of OMTs was constructed using Phylogeny. fr (https://www.phylogeny.fr/simple_phylogeny.cgi), and we found that CitOMT was categorized within the plant COMTs (Fig. 2b).

### Expression of flavonoid biosynthetic genes in citrus flavedo

**Figure 3:**
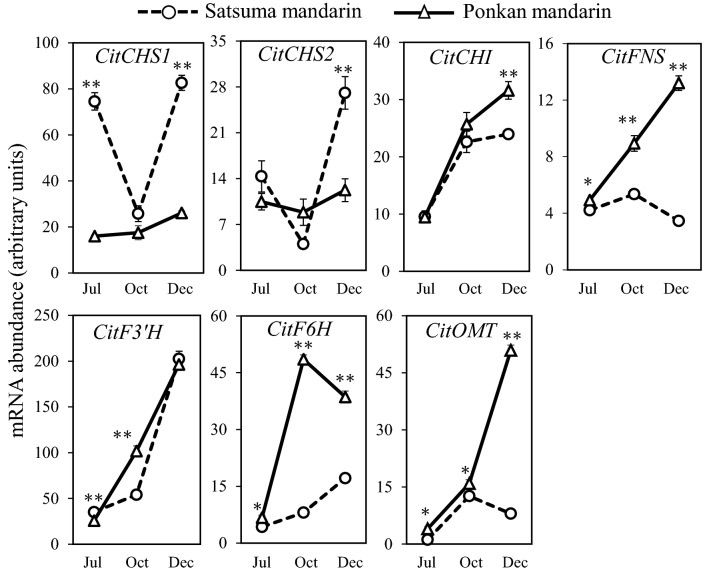
The expression of flavonoid biosynthetic genes in the flavedos of Satsuma mandarin and Ponkan mandarin during the fruit maturation. The mRNA levels were analyzed using TaqMan real-time quantitative RT-PCR. Real-time RT-PCR amplification of the 18S ribosomal RNA was used to normalize the expression of genes in same conditions. The results shown are means ± SE for triplicate samples. The significant difference between Satsuma mandarin and Ponkan mandarin is indicated by asterisks (*P < 0.05, **P < 0.01).

In this study, the expression of flavonoid metabolic genes (*CitCHS1*, *CitCHS2*, *CitCHI*, *CitFNS*, *CitF3*′*H*, and *CitF6H*), as well as *CitOMT* was investigated in the flavedos of Satsuma mandarin and Ponkan mandarin during fruit maturation. Sets of TaqMan probes and primers were designed based on the common sequences of the two varieties using Primer Express software (Supplementary Table S2). In Satsuma mandarin, the expression of *CitCHS1* and *CitCHS2*, which are related to the biosynthesis of chalcone, decreased to a low level at the transition stage, and then increased rapidly during maturation. In Ponkan mandarin, the expression of *CitCHS2* decreased at the transition stage, whereas the expression of *CitCHS1* increased slightly during fruit maturation. The expression levels of *CitCHS1* and *CitCHS2* in Satsuma mandarin was 3 times and 2.2 times higher than those in Ponkan mandarin at the mature stage, respectively. The expression levels of *CitCHI*, *CitFNS*, *CitF3*′*H*, *CitF6H*, and *CitOMT* increased gradually in the two citrus varieties during maturation. In Ponkan mandarin, the expression levels of *CitFNS*, *CitF6H*, and *CitOMT* were much higher than those in Satsuma mandarin during fruit maturation (Fig. 3).

### Enzyme activity of CitOMT in vitro

In order to investigate the function of *CitOMT* in citrus fruit, the cDNA of *CitOMT* was cloned into a pGEX-6P-1 vector, and the recombinant plasmid was transformed into *E. coli* strain XL1-Blue Competent Cells. Recombinant protein of CitOMT was obtained and affinity purified using PreScission Protease. The recombinant protein of CitOMT was detected as a single band by SDS-PAGE. The molecular weight of CitOMT was estimated 40.0 kDa, which was consistent with the estimated amino acid molecular weight.

**Figure 4:**
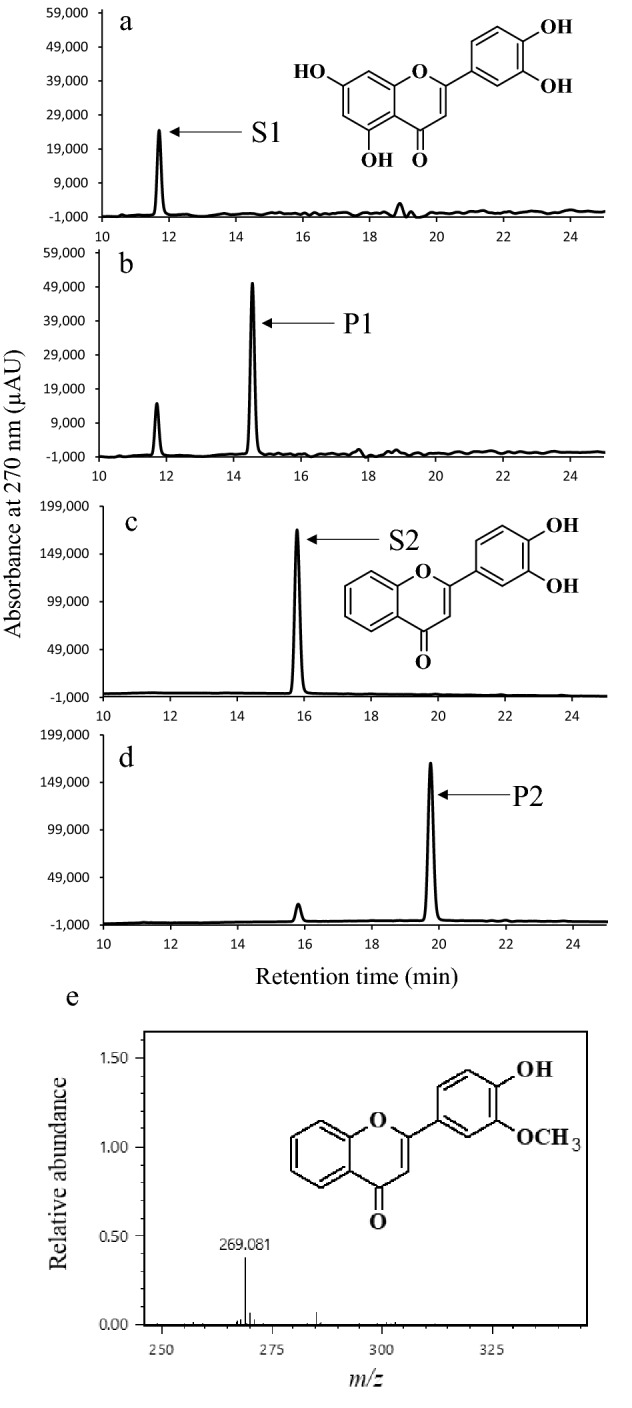
The enzyme activity of CitOMT in vitro. HPLC analysis of 3′,4′,5,7-tetrahydroxyflavone (**a**) and 3′,4′-dihydroxyflavone (**c**). HPLC analysis of methylated products of 3′,4′,5,7-tetrahydroxyflavone (**b**) and 3′,4′-dihydroxyflavone (**d**) catalyzed by CitOMT. Mass spectrometry spectrum of the methylated product of 3′,4′-dihydroxyflavone catalyzed by CitOMT (**e**). S1: 3′,4′5,7-tetrahydroxyflavone, S2: 3′,4′-dihydroxyflavone, P1: 3′,4′,5,7-tetrahydroxyflavone reaction product, P2: 3′,4′-dihydroxyflavone reaction product.

In order to investigate the substrate specificity of CitOMT, the recombinant protein was incubated with several flavonoids. As shown in Table 1, CitOMT methylated two flavones (3′,4′,5,7-tetrahydroxyflavone and 3′,4′-dihydroxyflavone), whereas it showed no OMT activity with flavanone or isoflavone. When 3′,4′,5,7-tetrahydroxyflavone was used as a substrate (peak S1 at 11.7 min, Fig. 4a), an *O*-methylated product of 3′,4′,5,7-tetrahydroxyflavone was detected (peak P1 at 18.9 min, Fig. 4b). When 3′,4′-dihydroxyflavone (peak S2 at 15.8 min, Fig. 4c) was used as a substrate, the peak of the *O*-methylated product was eluted (Peak P2 at 18.9 min, Fig. 4d). However, when CitOMT was reacted with flavones that only had a hydroxy group on the 4′-position, 7-position, or 7,8-position, no new products were detected (Table 1).

**Table 1:** Activities of CitOMT against a range of flavonoid substrates.

Group	Substrate	R	R_1_	R_2_	R_3_	R_4_	R_5_	Product^a^
Flavanone	3′-Hydroxyflavanone	OH	H	H	H	–	–	Not detected
	4′-Hydroxyflavanone	H	OH	H	H	–	–	Not detected
	Naringenin	H	OH	OH	OH	–	–	Not detected
	Hesperidin	OH	OCH_3_	OH	O-Rutinose	–	–	Not detected
Flavone	3′,4′-Dihydroxyflavone	OH	OH	H	H	H	H	Detected
	3′,4′,5,7-Tetrahydroxyflavone	OH	OH	OH	H	OH	H	Detected
	4′-Hydroxyflavone	OCH_3_	OH	OCH_3_	OCH_3_	OCH_3_	OCH_3_	Not detected
	7-Hydroxyflavone	H	H	H	H	OH	H	Not detected
	7,8-Dihydroxyflavone	H	H	H	H	OH	OH	Not detected
Isoflavone	Daidzein	OH	–	–	–	–	–	Not detected

^a^‘Detected’ or ‘Not detected’ indicates whether there was a new peak detected by HPLC analysis.

To further confirm the role of CitOMT, the reaction product of 3′,4′-dihydroxyflavone was analyzed by Direct Analysis in Real Time (DART) MS on positive mode. Mass spectrometry showed that the *O*-methylated product of 3′,4′-dihydroxyflavone (Peak P2 at 18.9 min, Fig. 4d) had a parent ion peak [M + H]^+^ at an *m/z* of 269.081, and its formula was calculated as C_16_H_13_O_4_. The results of mass spectrometry analysis suggested that the *O*-methylated product of 3′,4′-dihydroxyflavone (P2) was a mono-methylated flavone (Fig. 4e). In addition, because the hydroxy group on its 4′-position cannot be methylated by CitOMT (Table 1), it was indicated that CitOMT may function to methylate the 3′-hydroxy group of flavones in vitro.

## Discussion

Citrus plants are a rich source of flavonoids, and the accumulation of flavonoids is closely related to the appearance, taste, as well as the nutritional values of the fruit. The major flavonoids accumulated in citrus fruit are divided into two groups, flavanone glycosides, such as naringin, hesperidin, neo-hesperidin, and PMFs, such as, nobiletin, sinensetin, and tangeretin^35^. It is well known that the accumulation of flavonoids in citrus fruit depends on several factors, including the genetic origin, maturity stage, and the different parts of the fruit (flavedo, albedo, seeds, Supplementary Figure S1). Moreover, the composition of flavonoids varies greatly among different citrus species^24,36,37^. In the present study, the accumulation of flavonoids was investigated in two citrus varieties, Satsuma mandarin and Ponkan mandarin. The results showed that there were significant differences in flavonoid composition between Satsuma mandarin and Ponkan mandarin (Fig. 1). In Satsuma mandarin, high amounts of flavanones were accumulated, while the contents of PMFs were extremely low, which accounted for less than 6% of the total flavonoid in the flavedo of mature fruit. In Ponkan mandarin, in contrast, high levels of PMFs were accumulated in the flavedo. In the flavedo of mature fruit, the PMFs contents in Ponkan mandarin accounted for more than 53% of total flavonoid. In Ponkan mandarin, four kinds of PMFs, sinensetin, nobiletin, tangeretin and heptamethoxyflavone, were detected, and among them nobiletin was found to be the major PMF accumulated in the flavedo, followed by sinensetin, tangeretin, heptamethoxyflavone. In the mature fruit, the nobiletin content in Ponkan mandarin was approximately 13 times higher than that in Satsuma mandarin. In the study of Zohra et al., the accumulation of nobiletin and tangeretin was investigated in 11 citrus cultivars. The results revealed that there was a significant correlation between the accumulation of nobiletin and tangeretin in the flavedos of citrus fruit, and nobiletin tended to accumulate at higher level than tangeretin in the flavedos of the 11 citrus cultivars^38^.

To date, although flavonoid accumulation has been extensively reported in different citrus cultivars, the molecular mechanism regulating the biosynthesis of PMFs, especially nobiletin, in citrus fruit is still unclear. In the present study, to further elucidate the high accumulation of nobiletin in Ponkan mandarin, the expression of flavonoid biosynthetic genes (*CitCHS1*, *CitCHS2*, *CitCHI*, *CitFNS*, *CitF3*′*H*, *CitF6H*, and *CitOMT*) was investigated (Fig. 3). The results showed that the expression levels of genes that are responsible for PMF biosynthesis (*CitFNS*, *CitF3*′*H*, and *CitOMT*) were much higher in Ponkan mandarin than in Satsuma mandarin. The high expression levels of *CitFNS*, *CitF3*′*H*, and *CitOMT* contributed to the massive accumulation of nobiletin in the flavedo of Ponkan mandarin. In addition, the different expression levels of *CitFNS*, which a key gene that converts flavanones into flavones in plants, may lead to the distinct flavonoid composition between Satsuma mandarin and Ponkan mandarin. In Ponkan mandarin, the expression of *CitFNS* increased significantly during the maturation process, and the high expression level of *CitFNS* led to metabolic flux towards flavone synthesis. In Satsuma mandarin, in contrast, the expression of *CitFNS* increased with a small peak at the transition stage, and its expression level was much lower than that in Ponkan mandarin. The low expression level of *CitFNS* may limit the synthesis of flavones, and as a result high amounts of flavanones were accumulated in Satsuma mandarin.

OMTs that transfer the methyl group of SAM to the hydroxyl group of flavonoids are key enzymes for PMF biosynthesis. Plant OMTs are a large gene family, which are categorized into two types, COMT and CCoAOMT, according to their molecular weight and bivalent ion dependency. In plants, numerous OMT genes have been identified, and their functions have been extensively investigated in various plant species, such as Arabidopsis^39^, barley^40^, mango^41^, rice^42^, tomato^43^, and sweet basil^44^. In citrus, it was reported that 58 OMT genes existed and were unevenly distributed on the nine chromosomes of *Citrus sinensis*. Among them, 27 OMTs were predicted to be involved in the *O*-methylation of flavonoids from the DGE and qRT-PCR analysis^45^. To date, two OMTs, *CdFOMT5* and *CrOMT1*, have been isolated and their functions were characterized in citrus fruit. Recombinant proteins of CdFOMT5 and CrOMT1 exhibited high substrate specificity and regioselectivity. Recombinant CdFOMT5 demonstrated methylation activity for the 3-,5-,6-, and 7-hydroxyl groups of flavones in vitro^31^. Different from CdFOMT5, CrOMT1 is a CCoAOMT-like enzyme, and it had a strong preference for flavones with highest catalytic efficiency at the 6-and 8-hydroxyl groups of flavones in vitro^46^. In the present study, we isolated a novel OMT gene (*CitOMT*) from Satsuma mandarin and Ponkan mandarin, using the sequence of ROMT-9 as a query, which has been reported to have strict specificity for the 3′-hydroxy group of flavonoids^33^. In the phylogenetic analysis, it was shown that CitOMT was clustered into COMT, which is independent of a cation and known to have the enzymic activity for flavonoids^8–10^ (Fig. 2). In addition, multiple sequence alignment of CitOMT with other plant OMTs suggested that the amino acid sequence of CitOMT had the same conserved sequences including SAM binding residues and catalytic residues as other plants OMTs^5,6,34^, which indicates that CitOMT may possess *O*-methyltransferase activity with flavonoids in citrus fruit (Fig. 2).

Sequence analysis showed that CitOMT shared 53.8% and 23% identity with CdFOMT5 and CrOMT1 at the amino acid level, respectively. The relatively low identity levels indicated that the functions of CitOMT may be different from CdFOMT5 and CrOMT1 in citrus fruit. In the present study, to investigate the roles of *CitOMT* in citrus fruit, the cDNA of *CitOMT* was isolated from Ponkan mandarin, and cloned into a pGEX-6P-1 vector. A single band of the recombinant CitOMT protein was detected at approximately 40.0 kDa by SDS-PAGE. Functional analysis showed that the recombinant protein of CitOMT methylated two flavones (3′,4′,5,7-tetrahydroxyflavone and 3′,4′-dihydroxyflavone), whereas it had no activity with flavanones (3′-hydroxyflavanone, 4′-hydroxyflavanone, naringenin, hesperidin) and isoflavone (daidzein) in vitro. To further confirm the methylation position of CitOMT in flavones, the substrates, 4′-hydroxyflavone, 7-hydroxyflavone, 7,8-dihydroxyflavone, were also tested in vitro assays, and no new product was detected. These results suggested that CitOMT cannot methylate flavones at positions 4′, 7, or 8 in vitro. In addition, the *O*-methylated product of 3′,4′-dihydroxyflavone (Peak P2 at 18.9 min, Fig. 4d) was analyzed by DART MS, and the results showed that the *O*-methylated product of 3′,4′-dihydroxyflavone (P2) was a mono-methylated flavone (Fig. 4e). Because the hydroxy group on its 4′-position cannot be methylated by CitOMT (Table 1), it was indicated that CitOMT might have the function to methylate the 3′-hydroxy group of flavones in vitro.

**Figure 5:**
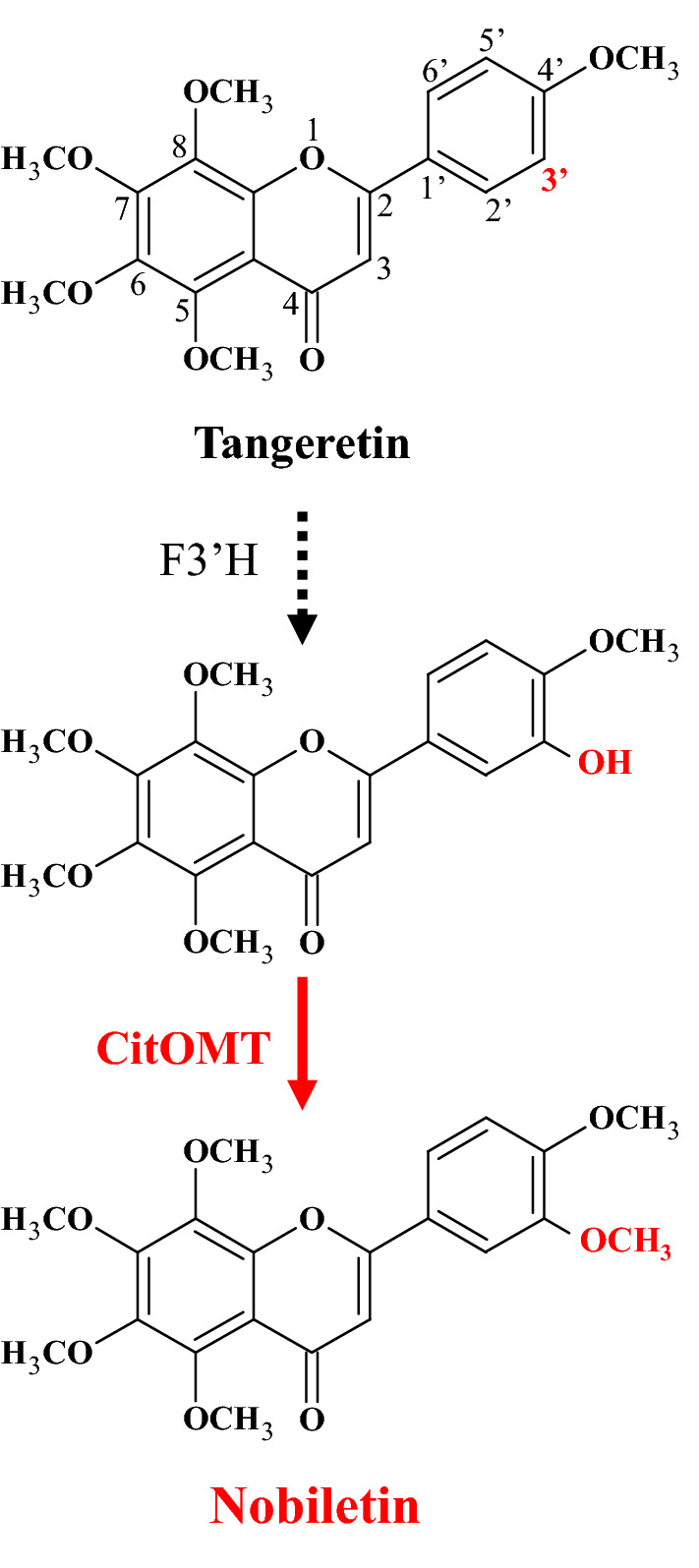
The proposed nobiletin biosynthetic pathway. The arrow with a dotted line indicates the estimated reaction from tangeretin. The arrow with a straight line indicates the reaction investigated in this study.

Nobiletin is a kind of polymethoxy flavone with six methoxy groups at the 3′,4′,5,6,7,8-positions. In the present study, functional analysis showed that CitOMT exhibited methylation activity to transfer a methyl group to the 3′-hydroxy group of flavones. Because methylation at 3′-position of flavone is vital for nobiletin biosynthesis, it was suggested that *CitOMT* was a key gene involved the nobiletin biosynthesis in citrus fruit. In addition, tangeretin has a similar structure with nobiletin, containing five methoxy groups at the 4′,5,6,7,8-positions. Both nobiletin and tangeretin were accumulated in the flavedos of citrus fruit, and the changes in the contents of these two flavonoids were similar during the maturation process (Fig. 1). Therefore, it was deduced that nobiletin may be biosynthesized from tangeretin catalyzed by CitF3′H and CitOMT in citrus fruit (Fig. 5).

In conclusion, the roles of a novel OMT gene (*CitOMT*) in nobiletin biosynthesis was investigated in two citrus varieties, Satsuma mandarin, which accumulates a low level of nobiletin, and Ponkan mandarin, which accumulates a high level of nobiletin. The results showed that the expression level of *CitOMT* in the flavedo of Ponkan mandarin was much higher than that in Satsuma mandarin during fruit maturation. In addition, functional analysis suggested that CitOMT was a key gene responsible for nobiletin biosynthesis in citrus fruit. Recombinant protein of CitOMT had methylation activity to transfer a methyl group to the 3′-hydroxy group of flavones in vitro, which is vital for nobiletin biosynthesis. The results presented in this study may contribute to elucidating the mechanism of nobiletin biosynthesis in the flavedo of Ponkan mandarin, which will provide strategies to improve flavonoid accumulation in citrus fruit.

## Methods

### Plant materials

Satsuma mandarin ‘Miyagawa-wase’ (*C. unshiu* Marc.) and Ponkan mandarin ‘Ohta Ponkan’ (*C. reticulata* Blanco) were grown at the Center for Education and Research in Field Sciences (Shizuoka, Japan). Flavedo was separated from sampled fruits, frozen in liquid nitrogen immediately, and kept at − 80 °C until use.

### Flavonoid analysis

Powdered flavedos were freeze dried. Portions (20 mg) were extracted by homogenization and ultrasonicating in 400 mL of DMSO:methanol (1:1, v/v) at room temperature. After centrifugation at 21,500 × *g* for 10 min, the supernatant was collected, and the remaining residue was extracted twice with 300 μL of the same solvent. In total, 1 mL of supernatant was filtered through a membrane filter, TORAST Disc (hole diameter: 0.22 μm, SHIMADZU GLC, Japan).

The high-performance liquid chromatography (HPLC) system consisted of a pump, autosampler, photodiode array detector, column oven (JASCO, Japan), and a YMC-UltraHT Pro C_18_ column (100 × 3.0 mm i.d. S-2 μm, 12 nm; YMC, Japan). The detector was monitored at 274, 310, 324, 338, and 362 nm. A two-solvent gradient system of 1% phosphoric acid (A) and acetonitrile-methanol (1:1, v/v) (B) was used. The gradient program consisted of three periods: (1) 0–33 min, 78% A, (2) 33–47.5 min, 16% A, (3) 47.5–75 min, 78% A. The flow rate was 0.6 mL min^−1^, the column was operated at 44 °C, and the sample injection volume was 10 μL. Standard flavonoids (eriocitrin, narirutin, naringin, hesperidin, rhoifolin, isorhoifolin, diosmin, sinensetin, nobiletin, tangeretin and heptamethoxyflavone) were purchased in FUJIFILM Wako Pure Chemical Corporation (Japan). The flavonoids were identified by comparing their retention times and UV spectra with those of authentic standards stored in a data processor. The concentration of each flavonoid was calculated from the integrated peak area of the sample and the corresponding standard. Each sample was replicated three times, and mean values and standard error were calculated.

### Gene expression

Total RNA was extracted from the flavedo of Satsuma mandarin and Ponkan mandarin according to the method described by Ikoma et al.^47^. The total RNA was purified using a RNeasy Mini Kit (Qiagen, Germany) and treated with DNase (Takara, Japan) digestion on the column. The cDNA was synthesized from 600 ng of purified RNA and a random hexamer primer at 37 °C for 60 min using TaqMan Reverse Transcription Regents (Applied Biosystems, USA).

Real-time PCR was performed to investigate the expression of *CitCHS1, CitCHS2*, *CitCHI*, *CitFNS*, *CitF3*′*H*, *CitF6H*, and *CitOMT.* TaqMan probes and sets of primers were designed based on the common sequences with Primer Express software (Supplementary Table S2, Applied Biosystems, USA). The reaction of real-time PCR was performed with cDNA template, 900 nM primers, and 250 nM TaqMan MGB probe. The thermal cycling conditions were 95 °C for 10 min followed by 40 cycles of 95 °C for 15 s and 60 °C for 60 s. The levels of gene expression were analyzed using ABI PRISM 7300 Sequence Detection System Software (Applied Biosystems, USA) and normalized with the result of 18S ribosomal RNA. Real-time PCR was performed in three replicates for each sample, and the mean values and the standard error were calculated.

### Expression and purification of recombinant CitOMT

To express the recombinant protein of CitOMT, cDNA from Ponkan mandarin was amplified by RT-PCR with set of primers (Supplementary Table S3). The cDNA fragments of *CitOMT*-coding gene were digested by BamHI and XhoI and purified using a GFX PCR DNA and Gel Band Purification Kit (GE Healthcare, Japan). The purified DNA fragment was ligated into the expression vector pGEX-6P-1 (Amersham Bioscience, UK), which had been digested with the same restriction enzymes. The constructed plasmid was transformed into *E. coli* strain XL1-Blue Competent Cells (Agilent Technology, Japan). For protein expression, 2 mL of overnight culture of the transformants harboring the gene of *CitOMT* was used to inoculate a 200 mL culture in a 2 × YT medium (5 g yeast extract, 8 g bacto-tryptone, and 2.5 g NaCl) to OD_600_ 0.8 at 37 °C with shaking. The expression and purification of recombinant protein of CitOMT were carried out using the method described by Kato et al.^48^. The expression of protein was induced by the addition of isopropyl-β-D-thiogalactoside (100 μM) at 27 °C for 17 h. The *E. coli* cells were collected by centrifugation at 3,300 × *g* for 10 min, and then resuspended in 20 mL of 1 × PBS stock solution of a GST Bulk Kit (GE Healthcare, Japan) containing 5 mM DTT. Suspensions containing the *E. coli* cells were lysed by sonication, and then 1% (v/v) of Triton X-100 was added and shaken on ice for 30 min. After centrifugation at 3,300 × *g* for 90 min, recombinant protein of CitOMT bonded to Glutathione Sepharose 4B (GE Healthcare) was washed twice with wash buffer [1 × PBS stock solution, 5 mM DTT, and 1% Triton X-100 (v/v)] and equilibrated twice with cleavage buffer [50 mM Tris–HCl, pH 7.0, 150 mM NaCl, 1 mM EDTA, 1 mM DTT, and 0.05% Triton X-100 (v/v)]. The recombinant protein was released using PreScission Protease (GE Healthcare, Japan) in cleavage buffer at 4 °C for 16 h. The recombinant protein was analyzed by SDS-PAGE using a 12.5% (v/v) polyacrylamide gel and WIDE-VIEW Prestained Protein Size Marker (Wako, Japan) using PhastSystem (Amersham Bioscience, US).

### Assay of enzyme activity

To investigate the enzymatic function of CitOMT from Ponkan mandarin, the purified recombinant protein was tested for its reaction with several flavonoids: 3′-hydroxyflavanone, 4′-hydroxyflavone, naringenin, and hesperidin as flavanones, 3′,4′-dihydroxyflavone, 3′,5,6,7,8-pentamethoxy-4′-hydroxyflavone^49^, 3′,4′,5,7-tetrahydroxyflavone, 7-hydroxyflavone and 7,8-dihydroxyflavone as flavones, and daidzein as an isoflavone, in the presence of *S*-adenosyl-l-methionine (SAM) as methyl donor. The reaction mixture consisted of 10 mM substrate, 500 mM SAM, 20 mM Tris–HCl (pH 7.0), 10% glycerol (v/v), 5 mM DTT, and 0.5 mg of purified CitOMT yielding a total volume of 500 μL. The reaction mixture was incubated at 30 °C with shaking for 1 h. The reaction solution was analyzed by HPLC. The HPLC system was the same with method described above. A two-solvent gradient system of 1% phosphoric acid (A) and acetonitrile-methanol (1:1, v/v) (B) was also used. The gradient program consisted of three periods: (1) 0–5 min, 78% A, (2) 5–22 min, 50% A, (3) 22–23 min, 16% A, (4) 23–28 min, 16% A, (5) 28–30 min, 78% A. The flow rate was 0.6 mL min^−1^, the column was operated at 44 °C. The reaction products were purified and analyzed by DART MS. The sample was injected directly to DART equipped with Cold Spray Ionization (CSI)^50^, and it was measured on positive mode. The stream of the sample vapor was directed in the zone between the DART-SVP ionization source (IonSense, Saugus, MA) and the inlet of the JMS-T100LP AccuTOF LC-plus 4G mass spectrometer (JEOL Ltd., Tokyo, Japan) with a time-of-flight mass spectrometer with resolving power of ≥ 10,000 (measured at nominal *m*/*z* = 609 according to FWHM definition). The ion source was operated with helium (purity > 99.99%) with flow rate of 3.5 L min^−1^. The DART internal heater was set at 300 °C. The voltage of the orifice 1 was set to 30 V for the experiment. The recording interval frequency was 0.4 s/spectrum.

### Statistical analysis

All values are shown as the mean ± SE for three replicates. The data were analyzed. Student’s *t*-test (*P* < 0.05 and *P* < 0.01) was used to compare the different varieties.

### Accession numbers of nucleotide sequence

The nucleotide sequences of the isolated *CitOMTs* were submitted to the DNA Data Bank of Japan (DDBJ) under the following accession numbers: LC516612 (Satsuma mandarin), LC616611 (Ponkan mandarin).

## Supplementary information

Supplementary information.
