# Supplementary material for: Expression and functional analysis of the nobiletin biosynthesis-related gene CitOMT in citrus fruit
Source: Sci Rep. 2020 Sep 17;10:15288. doi: 10.1038/s41598-020-72277-z (PMC7498457; doi:10.1038/s41598-020-72277-z)
Supplement: Supplementary file 1 — Supplementary information. [file 41598_2020_72277_MOESM1_ESM.pdf]

SUPPLEMENTARY MATERIALS

Expression and functional analysis of the nobiletin biosynthesis-related gene *CitOMT* in citrus fruit

Mao Seoka[1+], Gang Ma[1,2+], Lancui Zhang[2], Masaki Yahata[1,2], Kazuki Yamawaki[1,2], Toshiyuki Kan[3] & Masaya Kato[1,2*]

[1]Graduate School of Integrated Science and Technology, Shizuoka University, 836 Ohya, Suruga, Shizuoka, 422-8529, Japan

[2]Department of Bioresource Sciences, Faculty of Agriculture, Shizuoka University, 836 Ohya, Suruga, Shizuoka, 422-8529, Japan

[3]School of Pharmaceutical Sciences University of Shizuoka, 52-1 Yada, Suruga-ku, Shizuoka 422-8526, Japan

[+]These authors contributed equally: Mao Seoka and Gang Ma

*Corresponding author: Masaya Kato

Telephone: +81-54-238-4830 Fax: +81-54-238-4830

E-mail: kato.masaya@shizuoka.ac.jp

Supplementary Figure S1

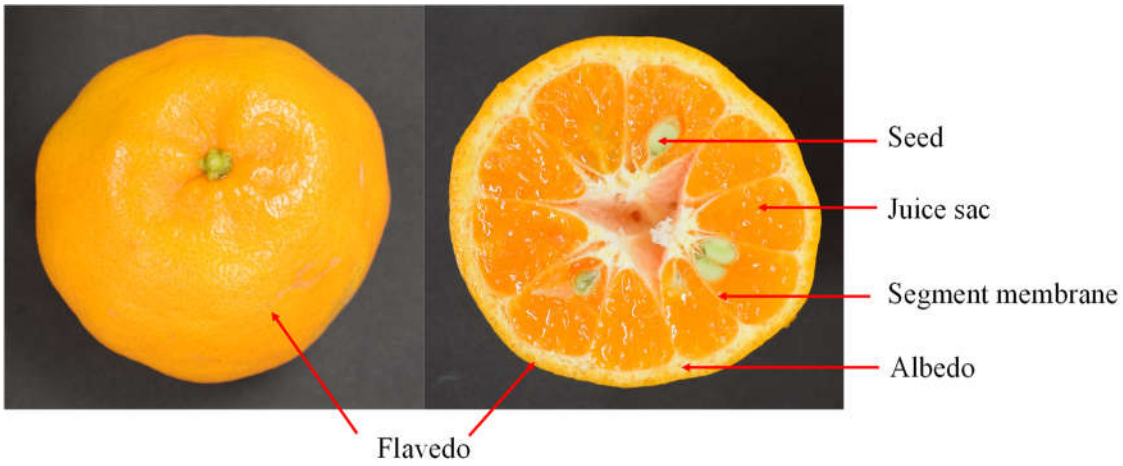

Supplementary Figure S1. The different parts of citrus fruit

Supplementary Table S1. Primer sequences used for the amplification of full length *CitOMT* gene (containing UTR)

cDNA		Primer sequence (5’→3’)
CitOMT	Forward	CCCCGAAAAAAGAAGGAAAAGAGGGAG
	Reverse	CAACTCTTTGCAACAACAATTCC

Supplementary Table S2. Sequences of primers and TaqMan probes used for real-time quantitative RT-PCR of flavonoid biosynthetic genes

cDNA		Primer sequence (5'→3')	TaqMan Probe
CitCHS1	Forward	CGTCTCATGATGTACCAACAAGGT	TTTTGCCGGTGGCACG
	Reverse	TGTTGTTCTCGGCTAGGTCTTTG	
CitCHS2	Forward	CCGGCGGCACTGTTCTT	CCTCGCTAAAGACTTG
	Reverse	AGCGCCCTTGTTGTTCTCA	
CitCHI	Forward	GAGTTCTTCAGAGACGTCGTCACA	TCCCTTTGAGAAATTC
	Reverse	GGCAAGATCATTGTCACCTTCA	
CitFNS	Forward	TGTGTTGTTTGTGAACCTTTGGT	ATGGGTAGGGACCCC
	Reverse	TCCAATGGGTTCTTCCAAATTT	
CitF3'H	Forward	CGTGGCGGCCCAATT	TGAAGATCCATGATTCCAA
	Reverse	GGAGGGCGGTTGGAGAA	
CitF6H	Forward	CGACCTCACTCTCGGACTCAA	CGCCACACGGATC
	Reverse	TTGGAGCAAGAGTGTGATGGTT	
CitOMT	Forward	TTCAGGATGCTCCAGCTTTTC	TGTCGAGCATGTTGGG
	Reverse	CCTTTGGAACACTAACAAACATGTCT	

Supplementary Table S3. Primer sequences used to make *CitOMT* construct

cDNA		Primer sequence (5'→3')
CitOMT	Forward	GCCCGGGATCCATGGGTTCAACCAGTTCAG
	Reverse	GCCCGCTCGAGTCAAGCACTCTTGAGAAAT

* The underlined sequences indicate the restriction enzyme sites.
